# *Aleurites moluccana* (L.) Willd. Leaves: Mechanical Antinociceptive Properties of a Standardized Dried Extract and Its Chemical Markers

**DOI:** 10.1155/2011/179890

**Published:** 2011-03-23

**Authors:** Nara L. M. Quintão, Christiane Meyre-Silva, Gislaine F. Silva, Carla S. Antonialli, Lilian W. Rocha, Ruth M. Lucinda-Silva, Angela Malheiros, Márcia M. Souza, Valdir Cechinel Filho, Tania M. B. Bresolin

**Affiliations:** Programa de Mestrado em Ciências Farmacêuticas, Núcleo de Investigações Químico-Farmacêuticas (NIQFAR), Universidade do Vale do Itajaí (UNIVALI), Rua Uruguai 458, 88302-202 Itajaí, SC, Brazil

## Abstract

Seeking to develop a new analgesic phytomedicine, a spray-dried extract (SDE) of *Aleurites moluccana* (L.) Willd. leaves was developed in scale up (5 kg). The SDE was standardized at 3% w/w in relation to the flavonoid 2′′-*O*-rhamnosylswertisin. The SDE batches were evaluated in relation to their physical, physiochemical, and pharmacological characteristics. The results demonstrated the reproducibility of the scale up SDE process which, when dosed orally, reduced carrageenan-induced mechanical hypernociception, with an ID_50_% of 443 mg/kg. Similar results were obtained with animals injected with complete Freund's adjuvant (CFA), in which SDE caused inhibition of 48 ± 4%. SDE was effective in preventing prostaglandin E2 (PGE2)-induced mechanical hypernociception (inhibition of 26 ± 10% and 33 ± 3%, at 250 and 500 mg/kg, respectively). Swertisin and 2′′-*O*-rhamnosylswertisin isolated from the own extract were effective in inhibiting the hypernociceptive response induced by carrageenan (70 ± 2% and 50 ± 5%, resp.). Furthermore, 2′′-*O*-rhamnosylswertisin was capable of significantly inhibiting the mechanical sensitization induced by CFA or PGE2, with inhibitions of 25 ± 3% and 94 ± 6%, respectively. These results suggest that the effects of SDE are related, at least in part, to the presence of these flavonoids.

## 1. Introduction

*Aleurites moluccana* (L.) Willd., *Euphorbiaceae*, popularly known as “Candlenut tree” or “Indian Walnut”, among other names, is a native plant to Malaysia, Polynesia, and the South Sea Islands, with wide distribution throughout the tropics. It is a medium-sized tree, up to 10 m tall, which grows abundantly throughout the south and southeast of Brazil (São Paulo to Rio Grande do Sul). It is used in folk medicine for the treatment of fever, inflammation, asthma, hepatitis, headache, gastric ulcer, and so forth. Its seeds are used as an antirheumatic and as a fertilizer [1, 2]. Previous studies have demonstrated that the dichloromethane fraction obtained from the leaves and bark of *A. moluccana *presents antiviral activity [3], as well as antibacterial effects against *Staphylococcus aureus* and *Pseudomonas aeruginosa* [4]. The methanol extract from the leaves of this plant exhibited hypolipidemic effects [5].

*A. moluccana *was introduced to Brazil in the 1980s as an ornamental tree and is an important source of the oil used in tannery [6]. There is a large area in Santa Catarina State (Brazil) with more than 6,000 trees that can furnish sufficient raw material for the pharmaceutical industry, and the culture of domesticated species has already begun. Studies conducted by our research group have demonstrated the performance of the tree after some types of cutting and have found that it develops well, requiring three to four months to germinate. The purpose of these studies is to assure the vegetable raw material source without interfering in the growth of the tree, thereby ensuring sustainable management of the biodiversity that culminates in the development of a herbal medicine [7]. These results are in agreement with previous studies, which observe that it can withstand both high and low pH values, poor soils, slopes, and annual rainfall of 6.4 to 42.9 dm [6].

Previous experimental studies conducted by our research group have also demonstrated that the hydroalcoholic extract of *A. moluccana leaves* and its hexane fraction exhibit antinociceptive effects, inhibiting acetic-acid-induced abdominal writhing in mice [8]. This activity may be related to the presence of 2′′-*O*-rhamnosylswertisin, a flavone *C*-glycoside, which caused 92 ± 4% inhibition when evaluated in the same model [9]. However, a more friendly technological extract needs to be developed, with a view to enabling industrial production of a herbal medicinal product, which must be obtained on a large scale and standardized in relation to a chemical marker, with stability, safety, and proven efficacy in preclinical and clinical studies, for further licensing by the regulatory agencies for marketing and purposes, under patent [7].

The aim of this work, therefore, is to obtain a reproducible spray-dried extract (SDE) in the scaleup, standardized in relation to a chemical marker, the flavonoid 2′′-*O*-rhamnosylswertisin (MW = 592 g), the major compound evidenced in the selected extract of *A. moluccana* leaves, and to evaluate the antinociceptive effect of the technological product, SDE, and two purified substances, 2′′-*O*-rhamnosylswertisin and swertisin, on different experimental models of pain in mice.

## 2. Materials and Methods

### 2.1. Collection of Plant Materials

Leaves of *A. moluccana *Willd. were collected in July 2007 in Tijucas, in the State of Santa Catarina, Brazil, and identified by Professor Dr. Ademir Reis (Department of Botany/UFSC, Florianópolis, SC, Brazil). A voucher specimen was deposited at the Barbosa Rodrigues Herbarium (Itajaí, SC, Brazil) under number VC Filho 001.

### 2.2. Preparation of Standardized Dried Extract

Three batches of dried extracts were prepared at Centroflora (Botucatu, SP, Brazil) on a pilot scale (5 kg) using extractive conditions optimized in previous works carried out by our group. 70 kg of dried herbal drug was macerated in a reactor for 5 days without stirring, with 700 L of 70 : 30 (v/v) ethanol : water solution at room temperature. The solution was then percolated, passed through a filter wheel (40 mm) then passed through Nutshell filter with filter paper. The solution was concentrated in Bernauer concentrator at 70°C under vacuum (400 mmHg), to obtain total solids of approximately 40%. The concentrate was mixed with about 25% (w/w total solids of concentrate) of colloidal silicon dioxide and dried in an industrial spray dryer (GEA Niro, Søborg, Denmark) with an inlet temperature of 165–180°C and an outlet temperature of 70–80°C.

The dried extracts were analyzed: morphological aspect by scaning electron microscopy (SEM) with magnification of 60x, color, taste/odor, moisture, pH (10% solution in water), aqueous solubility (concentration of 10%), and bulk density, according to the methodology described in the United States Pharmacopeia [10].

The extracts were standardized by high-performance liquid chromatography (HPLC), based on the marker content, 2′′-*O*-rhamnosylswertisin, using the methodology described below. 

#### 2.2.1. Isolation, Identification and Purity Determination of Swertisin and 2-*O*-Rhamnosylswertisin

**Figure 1:**
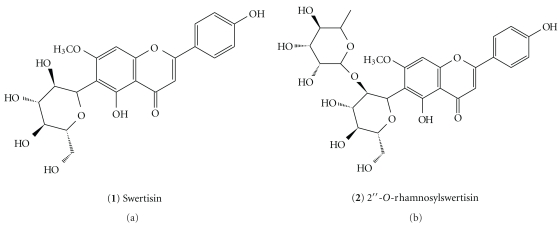
Chemical structure of (**1**) Swertisin and (**2**) 2′′-*O*-rhamnosylswertisin.

Milled air-dried leaves of *A. moluccana* (600 g) were extracted with methanol at room temperature for 7 days. The solution was then evaporated in a vacuum to obtain methanol crude extract (yield of 8.5%). Purification procedures (chromatographic column—CC) were conducted with crude methanol extract using silica gel 60 (70–230 mesh) and elution with increasing concentrations of methanol in dichloromethane to give nine fractions (I–IX). Fractions I and II were grouped and submitted to silica gel CC eluted with a mixture of dichloromethane and methanol in step-gradient mode, to yield swertisin **(1)** (Figure 1) in the form of a yellow, amorphous powder (20 mg), mp 242.6 to 243.4°C. The chemical structure was confirmed by NMR-^1^H and ^13^C, as previously reported [11, 12]. Similarly, fractions III and IV were grouped and submitted to silica gel CC eluted with a mixture of dichloromethane and methanol as eluent, with increasing polarity, followed by flash chromatography eluted with a mixture of dichloromethane and methanol (8 : 3) to yield 2′′-*O*-rhamnosylswertisin **(2) **(Figure 1) in the form of a yellow powder (490 mg). The chemical structure was confirmed by NMR-^1^H and ^13^C as previously reported [11, 13].

Swertisin (**1**) is an amorphous powder, mp 242.6–243.4°C. The isolated compound was confirmed by NMR spectroscopy using a Bruker AC-300 instrument, operating at 300 MHz for ^1^H and 75.5 MHz for ^13^C and using CD_3_OD as solvent. ^1^H NMR: 3.15–3.90 (m, 6 H, of Glc), 3.94 (3H, s, OCH_3_), 4.40 (1H, m, H1′′), 6.63 (1H, s, H3), 6.71 (1H, s, H8), 6.90 (2H, d, *j* = 8.8, H3′, H5′), 7.80 (2H, d, *j* = 8.8, H2′, H6′). ^13^C NMR: 55.9 (OCH_3_), 63.3 (C6′′), 71.4 (C4′′), 72.1 (C1′′), 74.6 (C3′′), 80.9 (C2′′), 83.2 (C5′′), 96.9 (C3), 101.4 (C8), 101.5 (C10), 111.8 (C6), 116.8 (C3′, C5′), 121.8 (C1′), 128.7 (C2′, C6′), 157.5 (C5), 161.6 (C4′), 162.5 (C9), 164.4 (C1), 164.5 (C7), and 182.8 (C4).

2′′-*O*-rhamnosylswertisin (**2**) an amorphous powder, mp 260.5–261.0°C. NMR spectroscopy (CD_3_OD). ^1^H NMR: 0.65 (3H, d, *J* = 6.0, H6′′′), 3.10–3.90 (m, 6H, of Glc, 5H rham) 3.96 (3H, s, OCH_3_), 4.55 (1H, m, H1′′), 5.11 (1H, m, H1′′′), 6.67 (1H, s, H3), 6.76 (1H, s, H8), 6.93 (2H, d, *j* = 9.0, H3′, H5′), 7.89 (2H, d, *j* = 9, H2′, H6′). ^13^C NMR: 18.0 (C6′′′), 55.9 (OCH_3_), 62.4 (C6′′), 64.4 (C5′′′), 70.0 (C4′′), 72.1 (C2′′′), 72.2 (C3′′′), 72.5 (C4′′′), 73.6 (C1′′), 76.9 (C3′′), 79.0 (C2′′), 81.8 (C5′′), 102.3 (C3), 103.1 (C1′′′), 103.7 (C8), 103.9 (C10), 111.1 (C6), 117.8 (C3′, C5′), 121.9 (C1′), 129.7 (C2′, C6′), 159.2 (C5), 159.5 (C4′), 165.5 (C9), 165.0 (C1), 167.0 (C7), and 184.0 (C4).

MS analysis was performed in the LC-MS/MS system: SCL-10AVP system controller (Shimadzu), LC-10ADVP solvent delivery system and an FCV-10ALVP valve (Shimadzu Corp., Kyoto, Japan) with a Micromass Quattro LC mass spectrometer (Waters Corp., Milford, MA) in tandem) by infusion of the compound added to a solvent flow of 0.6 mL/min, split 1 : 3, acetonitrile : 50 mM  ammonium acetate (90 : 10, v/v). This was brought into the MS via an ESI source and measured in the negative ion mode. Nebulization was achieved using nitrogen gas at a pressure of 50 L/h. The capillary temperature was set at 350°C. The ESI-MS spectrum recorded in the negative ion mode showed a [M-H] + peak at m/z 591, which was in agreement with the expected molecular weight of 592 (C_28_H_32_O_14_) for 2′′-*O*-rhamnosylswertisin. MS/MS analysis showed a loss of 146 mass units (peak at m/z 445), corresponding to the loss of rhamnosyl moiety. Peaks were also observed at m/z 427 and m/z 324. It should be pointed out that this is the first report on the MS analysis of this compound.

#### 2.2.2. Quantitative Analysis of 2′′-*O*-Rhamnosylswertisin Flavonoid in Dried Extract

The HPLC system consisted of a Waters 600 pump (Milford, Mass, USA); 2996 PDA detector, automatic 717 plus injector, in line degasser AF, and Millennium Empower software. The injections (20 *μ*L) were carried out on a C8 X-Bridge 150 × 4,6 mm (5 um) (Waters, Taunton, Mass, USA). The mobile phase consisted of a gradient A (acetonitrile) and B (acidified water pH 3.54 with acetic acid) of 10 : 90 (A : B) (0 min), 25 : 75 (20 min), and 10 : 90 (30 min), maintaining this composition until 40 min, and a flow rate of 0.5 mL·min^−1^. The analysis was monitored at 338 nm and the column oven fit to 30°C.

All solvents were HPLC grade (Tedia, Fairfield, Ohio, USA) and were degassed by ultrasonic bath (Unique, Santo Amaro, São Paulo, Brazil). The water was purified using a Milli-Q system (Millipore, Billerica, Mass, USA). All solutions were filtered through 0.45 *μ*m membranes (Millipore, Billerica, Mass, USA).

#### 2.2.3. Pharmacological Studies

**Animals**

Female Swiss mice (25–30 g), obtained from the University of Vale do Itajaí (UNIVALI, Itajaí, Brazil), were used throughout this study. Female mice were chosen to investigate mechanical hypernociception based on the literature data, which indicates that females are more susceptible to developing chronic nociception [14, 15]. The animals were housed under conditions of optimum light, temperature, and humidity (12 h light-dark cycle, 22 ± 1°C, 60 to 80% humidity), with food and water provided *ad libitum*. All procedures used in the present study followed the “Principles of Laboratory Animal Care” from NIH publication no. 85-23 and were approved by the Animal Ethics Committee of UNIVALI (Protocol numbers 416/2008 UNIVALI). The number of animals and the intensity of noxious stimuli used were the minimum necessary to demonstrate consistent effects. The number of animals per group was 5–8.

**Carrageenan-Induced Mechanical Hypernociception**

To induce inflammatory pain, the mice received an i.d. injection of 50 *μ*L of carrageenan (300 *μ*g/paw) under the surface of the right hindpaw [14]. To assess the systemic effect of the drug treatment, the mice received* A. moluccana* SDE (batch 1, 2, or 3; 125–500 mg/kg, p.o.), 2′′-*O*-rhamnosylswertisin (30 mg/kg; 50.6 *μ*mol/kg, p.o.), swertisin (30 mg/kg, 67.2 *μ*mol/kg, p.o.), indomethacin (0.1–5 mg/kg, p.o.), or vehicle (10 mL/kg, 0.9% NaCl solution), 1 h before carrageenan injection. The mechanical hypernociception of all the groups was assessed by means of von Frey filaments (VFH), for up to 48 h after carrageenan administration, as described below.

**CFA-Induced Mechanical Hypernociception**

In an attempt to evaluate the preventive effect, the mice were pretreated orally with *A. moluccana* SDE (batch 1; 125–500 mg/kg), 2′′-*O*-rhamnosylswertisin (10 mg/kg; 16.86 *μ*mol/kg, p.o.), indomethacin (10 mg/kg, p.o.), or vehicle (10 mL/kg, 0.9% NaCl solution). 1 h afterwards, they received an i.d. injection of complete Freund's adjuvant (CFA) (1 mg/mL heat-killed and dried *Mycobacterium tuberculosis*; each mL of vehicle contained 0.85 mL paraffin oil plus 0.15 mL mannide monooleate; 20 *μ*L/paw) in the right hindpaw [16]. Mechanical hypernociception was evaluated for up to 48 h after CFA injection.

**Mechanical Hypernociception Induced by PGE_2_**

To evaluate the mechanical hypernociception, mice were treated with* A. moluccana* SDE (batch 1; 125–250 mg/kg, i.p.), 2′′-*O*-rhamnosylswertisin (10 mg/kg; 16.86 *μ*mol/kg, p.o.), morphine (5 mg/kg, s.c), or vehicle (10 mL/kg, 0.9% NaCl solution), 30 min before the i.d. injection of prostaglandin E_2_ (PGE_2_) (0.1 nmol/paw) [16]. The mechanical hypernociception was measured with VFH, as described below, at different time points after the i.d. injection of PGE_2_.

**Von Frey Hair-Induced Hindpaw Withdrawal Response**

To evaluate mechanical hypernociception, the mice were individually placed in clear Plexiglas boxes (9 × 7 × 11 cm) on elevated wire mesh platforms, to allow access to the ventral surface of the right hindpaw. The withdrawal response frequency was measured following 10 applications (duration of 1 s each) of von Frey hairs (VFH, Stoelting, Chicago, Il, USA). The stimuli were delivered from below to the plantar surface of the right hindpaw. The animals were acclimatized for 30 min before behavioural testing, and mechanical hypernociception was evaluated at several time points. A VFH of 0.6 g produces a mean withdrawal frequency of about 15%, which is considered to be an adequate value for the measurement of mechanical hypernociception [14, 17]. Therefore, 0.6 g VFH was used throughout this study. To determine the basal mechanical thresholds, all the groups were evaluated before the test.

**Drugs and Reagents**

The following drugs and reagents were used: CFA (Sigma Chemical Company, St. Louis, Mo USA); carrageenan, PGE_2_ (Fluka Riedel-de Haën, Seelze, Germany); indomethacin (DEG Importação de Produtos Químicos Ltda). All the other reagents and solvents used were of analytical grade.

**Statistical Analysis**

The results are presented as the mean ± S.E.M. of 5 to 7 animals, except for the ID_50_ values (i.e., the dose of *A. moluccana* DE that reduced the hypernociceptive responses by 50% relative to the control values), which are presented as means accompanied by their respective 95% confidence limits. The ID_50_ values and the percentages of inhibition were based on AUC (area under curve), calculated using the entire time course of each experiment and reported as mean ± S.E.M. of inhibitions obtained for each individual experiment. Statistical comparison of the data was performed by two-way analysis of variance (ANOVA), followed by Bonferroni's *post hoc* test and by one-way ANOVA and Dunnett's *post hoc* test. *P*-values lower than 0.05 (*P* < 0.05 or less) were considered significant.

## 3. Results

### 3.1. Isolation, Identification, and Purity Determination of Flavonoids

The phytochemical procedures conducted with the crude methanol extract obtained from *A. moluccana* leaves using silica gel CC and flash chromatography led to the isolation and identification of two main flavone C-glycosides, swertisin (**1**) and 2′′-*O*-rhamnosylswertisin (**2**) (Figure 1). Although the spectral data has been previously published [13], this is the first report on the MS analysis of this compound.

**Figure 2:**
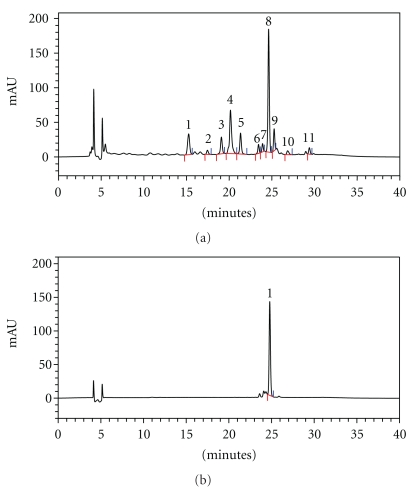
(a) Typical chromatogram of *A. moluccana* dried extract; (b) 2′′-*O*-rhamnosylswertisin chromatogram.

Purity analysis was performed by means of HPLC, and at 338 nm, 2′′-*O*-rhamnosylswertisin was identified with a 24.5 min retention time and swertisin with a 25.5 min retention time Figure 2(a). After integration of all the peaks at 338 nm, a purity >95% of flavonoids was calculated, Figure 2(a).

### 3.2. Preparation of Standardized Dried Extract

**Figure 3:**
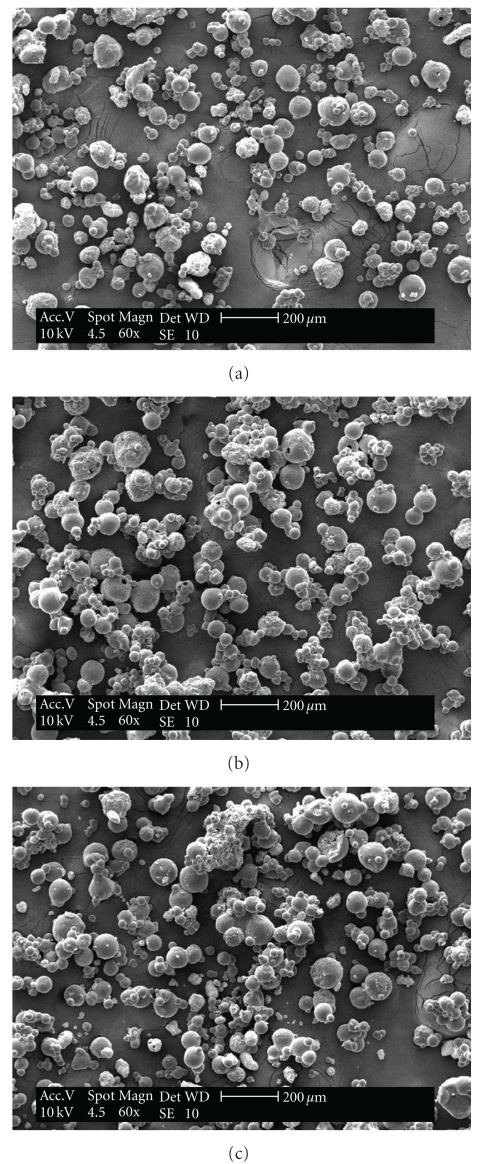
Scanning electronic microscopy of dried extracts of *A. moluccana* produced in pilot scale. (a) batch 1; (b) batch 2; (c) batch 3.

The *A. moluccana* dried extract batches obtained in pilot scale by spray drier showed similar physical and chemical characteristics, Table 1. The dried extracts are characterized as fine and hygroscopic powder, spherical particles with a smooth surface, greenish brown in color, with approximately 3% moisture (Figure 3). The 2′′-*O*-rhamnosylswertisin content of the extracts was similar, and the physical and chemical results were in agreement with the data obtained in previous studies to optimize the extraction process and biological activity *in vivo*. 

The chromatographic profile of dried extract by HPLC showed the presence of various flavonoid compounds including 2′′-*O*-rhamnosylswertisin (the major component, peak 8) and swertisin (peak 9), Figure 2(a).

### 3.3. Pharmacological Studies

**Figure 4:**
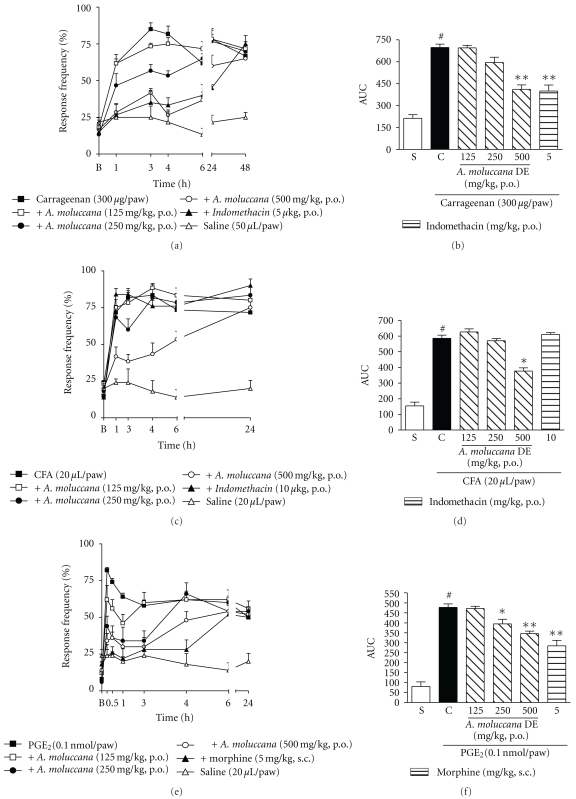
Effects of* A. moluccana* DE (batch 1; 125–500 mg/kg, p.o.) in mechanical hypernociception induced by i.d. injection of ((a) and (b)) carrageenan (300 *μ*g/paw), ((c) and (d)) CFA (20 *μ*L/paw), or ((e) and (f)) PGE_2_ (0.1 nmol/paw) in mice. Each group represents the mean of 5 to 8 animals, and the vertical lines indicate the SEM. Significantly different from the control values *P < 0.05 and significantly different from saline group ^#^ *P* < 0.001 (two-way ANOVA followed by Bonfferroni's *post hoc* test and one-way ANOVA followed by Dunnett's *post hoc* test).

Figures 4(a) and 4(b) demonstrate that i.d. injection of carrageenan (300 *μ*g/paw) significantly reduced the mechanical sensitivity threshold (*P* < 0.05), when compared to the response rate of the control group with animals injected with saline solution and basal thresholds. The oral treatment with *A. moluccana* (500 mg/kg) was able to reduce the mechanical hypernociception induced by i.d. injection of carrageenan, with inhibition of 36 ± 4% and ID_50_% of 443 (400–490) mg/kg. Indomethacin, a non steroidal antiinflammatory drug used as a positive control, was also capable of inhibiting the mechanical sensitization induced by carrageenan, with inhibition of 38 ± 5%.

Significant results were obtained with animals injected with CFA, where *A. moluccana* SDE (batch 1; 500 mg/kg, p.o.) reduced the hypernociceptive threshold for up to 6 h after CFA paw injection, with inhibition of 48 ± 4% at a dose of 500 mg/kg, see Figures 4(c) and 4(d). The nonsteroid antiinflammatory drug indomethacin (10 mg/kg, p.o.), dosed orally, did not interfere with the hypernociceptive threshold induced by CFA in mice.

As illustrated in Figures 4(e) and 4(f), i.d. injection of PGE_2_ (0.1 nmol/paw) into the mouse hindpaw produced prominent mechanical hypernociceptive effects lasting for up to 24 h, as indicated by a marked increase in baseline values in response to 0.6 g VFH stimulation. *A. moluccana* DE (batch 1; 125 to 500 mg/kg, p.o., 1 h) proved to be effective in significantly preventing PGE_2_-induced mechanical hypernociception up to 1 h after the irritant agent injection, Figures 4(e) and 4(f). The percentages of reduction were 26 ± 10% and 33 ± 3%, for 250 and 500 mg/kg, respectively. Morphine (5 mg/kg, s.c.), the drug used as positive control, reduced the hypernociceptive response induced by PGE_2_, with inhibition of 48 ± 6%, Figure 4(f).

**Figure 5:**
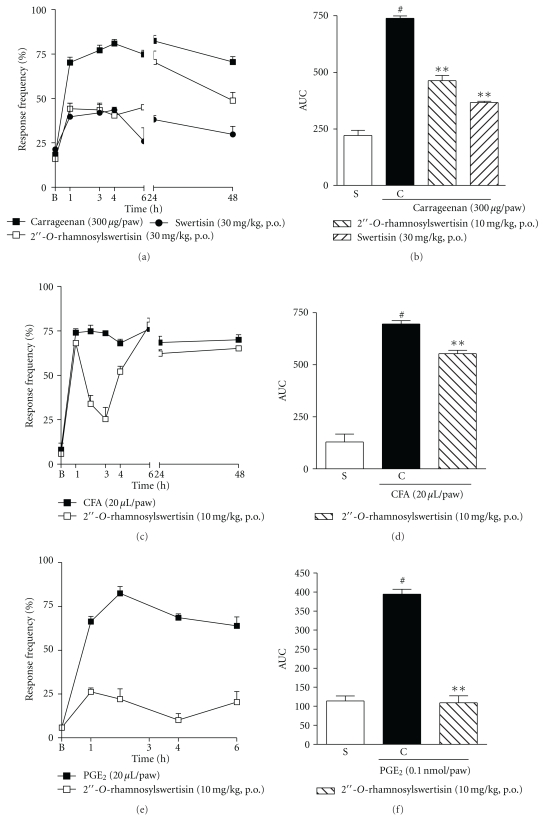
((a) and (b)) Effects of the isolated compounds 2′′-*O*-rhamnosylswertisin and swertisin, administered orally (30 or 10 mg/kg, 1 h before) in mechanical hypernociception induced by i.d. injection of ((a) and (b)) carrageenan (300 *μ*g/paw), ((c) and (d)) CFA (20 *μ*L/paw) or ((e) and (f)) PGE_2_ (0.1 nmol/paw) in mice. Each group represents the mean of 5 to 8 animals, and the vertical lines indicate the SEM. Significantly different from the control values *P < 0.05 and **P < 0.01 and significantly different from the saline group ^#^ *P* < 0.001 (two-way ANOVA followed by Bonfferroni's *post hoc* test and one-way ANOVA followed by Dunnett's *post hoc* test).

The antinociceptive effect of the isolated markers, swertisin and 2′′-*O*-rhamnosylswertisin (30 mg/kg, p.o., 1 h before), was also investigated in the carrageenan-induced mechanical hypernociception model in mice. Figures 5(a) and 5(b) demonstrate that both swertisin and 2′′-*O*-rhamnosylswertisin were effective in inhibiting the hypernociceptive response, when compared with the control group, with inhibitions of 70 ± 2% and 50 ± 5%, respectively. Furthermore, 2′′-*O*-rhamnosylswertisin was capable of significantly inhibiting the mechanical sensitization induced by CFA or PGE_2_, with inhibitions of 25 ± 3% and 94 ± 6%, respectively, see Figures 5(c) and 5(d). 2′′-*O*-rhamnosylswertisin accounts for 3% of the dried extract obtained from *A. moluccana*, as evidenced by HPLC, see Figure 2. 

**Figure 6:**
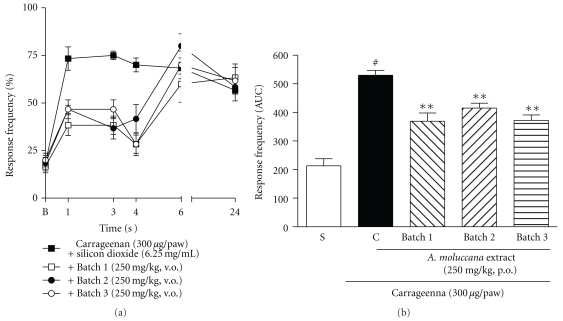
((a) and (b)) Effects of the* A. moluccana* extract batch 1, 2, or 3, administered orally (250 mg/kg, 1 h before), in the mechanical hypernociception induced by i.d. injection of carrageenan (300 *μ*g/paw) in mice. Each group represents the mean of 5 to 8 animals, and the vertical lines indicate the SEM. Significantly different from the control values **P < 0.01 and significantly different from the saline group ^#^ *P* < 0.001 (two-way ANOVA followed by Bonfferroni's *post-hoc* test and one-way ANOVA followed by Dunnett's *post-hoc* test).

Finally, when the dried extract produced in the scaleup was tested, all three batches had the same effect when compared with the previous studies, Figures 6(a) and 6(b). It is important to mention that the results obtained with the dose of 250 mg/kg presented in Figure 4(a) are significantly different from the control group, although the AUC representation did not demonstrate the same. 

## 4. Discussion

This paper describes the isolation, purification, characterization, and pharmacological effects of the chemical marker 2′′-*O*-rhamnosylswertisin and swertisin from leaves of *A. moluccana*. Although these flavonoids have been found in other plants, they are considered rare in higher plants and have not been described for plants belonging to the *Euphorbiaceae* family. These markers are important for the quality assurance of each step in the phytomedicines development and also to elucidate its pharmacological activity. 

The methodology of SDE preparation in the pilot scale shows reproducibility and feasibility for use in extract production on an industrial scale. Also, the HPLC-developed method allowed the standardization of the extract for the future development of a phytomedicine. 

The drugs used to treat chronic inflammatory conditions are not as effective and display pharmacological effect acting only on the inhibition of production of prostanoids but not interfering with other important elements of the cascade of inflammation. Thus, the pharmacological validation of natural products with established use in folk medicine in these types of conditions is very important. The pharmacologic effect of SDE and the markers was evaluated in a model of mechanical hypernociception induced by important flogistic agents such as carrageenan, PGE_2_, and CFA. Our study initially demonstrated the effect of SDE in the mechanical hypernociception induced by i.d. injection of carrageenan in mice. Carrageenan is an inflammatory agent that is largely used as pharmacological tool for investigating inflammatory hyperalgesia in rats and mice [18]. When injected intradermally on the plantar surface of animal's hindpaw, it induces an inflammatory process associated with hyperalgesia [19]. Tissue injury originating after the injection of carrageenan involves the release of different chemical mediators such as PGE_2_ [20], mast cells products histamine and serotonin [21], neuropeptides [22], and cytokines (TNF*α* and IL-1*β*) [23], among others [23]. This effect was similar when compared with animals treated with indomethacin. This result suggests that SDE of *A. moluccana* may be interfering with different pathways involved in inflammatory pain signaling.

The effect of SDE against inflammatory hypernociception induced by i.d. injection of CFA and PGE_2_ was also investigated. The CFA model has significant proximity with chronic inflammatory diseases in humans, such as rheumatoid arthritis. The inflammatory response induced by CFA develops rapidly and can persist for several weeks or months [14]. The hypernociceptive response is mediated by local sensitization of nociceptors and also involves immune response and changes in the central nervous system [24]. It is now well established that inflammatory hypernociception, such as that induced by PGE_2_, depends on the activation of signalling pathways, which require neuronal activation of PKA in neurons, downstream of the second messenger cAMP [25]. Moreover, there many studies suggesting that the PKC pathway also participates in nociceptor sensitization, induced by nociceptive mediators, such as adrenaline, endothelins, and bradykinin, as well as by more general inflammation.

These pharmacological data reinforce the fact that part of the antihypernocicepive effect observed with dried extract obtained from *A. moluccana* is probably due to the action of the isolated compounds 2′′-*O*-rhamnosylswertisin and swertisin. The role of flavonoids in the effects of *A. moluccana*, modulating the nociceptive threshold, can be hypothesized, since it has been extensively reported that this class of compounds is responsible for the antiinflammatory and anti-nociceptive effects of several herbal products, such as rutin, quercetin, and myricitrin, among others in animal models of pain and acute inflammation [26–28]. In addition, several flavonoids, including quercetin and myricitrin are also effective in reducing hypernociception in models of chronic inflammatory pain [29–31].

Previous studies conducted with the flavonoid swertisin revealed its potential as an agent that stimulates insulin secretion presenting antihyperglycemic effect [32], but no analgesic studies with this flavonoid have yet been conducted; except for tge work in [33] done on the flavonoid-enriched fraction from *Cayaponia tayuya* roots which included swertisin. 

Recent studies conducted at our laboratories have indicated the absence of toxicological effects of this extract in two different animal species (unpublished results). In addition, no cases of intoxication by this *Aleurites *species were found in the literature [34].

It is also suggested that dried extract of *A. moluccana*, dosed systemically, may be acting on the peripheral and/or on the central pathways of pain control. The mechanisms through which* A. Moluccana*, and its marker 2′′-*O*-rhamnosylswertisin, exert their anti-hypernociceptive actions are currently unclear and require further investigation; however, this could well constitute a new and attractive alternative for the management of inflammatory pain in humans using natural product. In view of the need for new safe and effective therapies, and taking into account the adverse effects associated with the drugs currently used, *A. moluccana *represents an important and promising source of herbal medicine for the treatment of pathologies for which no efficacious treatment exists, such as chronic pain.

## 5. Conclusion

The standardized dried extract of *A. moluccana* with 3% of the marker 2′′-*O*-rhamnosylswertisin was obtained in a reproducibility scaleup method. Furthermore, the extract showed potential antinociceptive effects in an experimental model of inflammatory mechanical sensitization, using different inductor agent, such as *λ*-carrageenan, CFA, or PGE_2_ in mice. The biological effect of the extract may be attributed, at least in part, to the marker 2′′-*O*-rhamnosylswertisin and to the other flavonoid, swertisin, present in minor amounts. These promising results support further studies aimed at developing a new herbal medicine based on a tree used in traditional folk medicine, contributing to the treatment of painful processes.

## Figures and Tables

**Table 1:** Physical and chemical characteristics of dried extracts of *Aleurites moluccana* produced in pilot scale.

Parameters	Dried extract batch		
	1	2	3
Aspect	Fine, hygroscopic powder	Fine, hygroscopic powder	Fine, hygroscopic powder
Color	greenish brown	greenish brown	greenish brown
Taste/odor	characteristic	characteristic	characteristic
Moisture (%)	2.6	3.0	3.4
Average size (*μ*m)	120.54	151.12	112.69
pH	4.95	4.76	4.77
Bulk density (g/mL)	0.6386	0.5757	0.6152
2′′-*O*-rhamnosylswertisin content (% w/w)	3.01	2.76	2.91
